# Supplementary material for: Copy Number Profiles of Prostate Cancer in Men of Middle Eastern Ancestry
Source: Cancers (Basel). 2021 May 14;13(10):2363. doi: 10.3390/cancers13102363 (PMC8153627; doi:10.3390/cancers13102363)
Supplement: Supplementary file 1 [file cancers-13-02363-s001.zip › cancers-1141750 - supple.- revised version/Table S1.pdf]

Sample	Ancestry	Age at treatm	Pre-treatmen	T category	Diagnostic Gl	IDC or Cribifo	ISUP Grade	Treatment	NKX3-1 FISH :	MYC FISH sta	Has SNP array
PCa1	Mid. East	75	11.21	T3a	3+4	FALSE	2	RP	0	0	Yes
PCa2	Mid. East	65	6.33	T3	4+3	TRUE	3	RP	-1	0	Yes
PCa3	Mid. East	62	6.86	T2c	3+4	FALSE	2	RP	0	0	Yes
PCa4	Mid. East	66	17.37	T3a	3+3	FALSE	1	RP	-1	0	Yes
PCa5	Mid. East	66	12	T3b	3+4	FALSE	2	RP	0	0	Yes
PCa6	Mid. East	65	13	T3b	4+3	TRUE	3	RP	0	0	Yes
PCa7	Mid. East	71	33.8	T3b	3+4	FALSE	2	RP	0	0	Yes
PCa8	Mid. East	59	NA	T2c	3+3	FALSE	1	RP	0	0	Yes
PCa9	Mid. East	67	6.7	T2c	3+3	FALSE	1	RP	0	0	Yes
PCa10	Mid. East	69	5.9	T2c	4+3	FALSE	3	RP	0	0	Yes
PCa11	Mid. East	60	6.4	T2c	3+3	FALSE	1	RP	0	0	Yes
PCa12	Mid. East	66	6.3	T2c	3+3	FALSE	1	RP	0	0	Yes
PCa13	Mid. East	68	13.2	T2a	3+4	FALSE	2	RP	0	0	Yes
PCa14	Mid. East	62	4.5	T3b	3+4	FALSE	2	RP	NA	NA	Yes
PCa15	Mid. East	73	5.6	T2a	3+3	FALSE	1	RP	NA	NA	Yes
PCa16	Mid. East	73	34.9	T3b	3+4	TRUE	2	RP	NA	NA	Yes
PCa17	Mid. East	67	6.29	T3a	3+4	FALSE	2	RP	-1	0	Yes
PCa18	Mid. East	70	74.07	T3a	4+4	FALSE	4	RP	0	0	Yes
PCa19	Mid. East	66	12.8	T2	3+4	FALSE	2	RP	0	0	Yes
PCa20	Mid. East	72	18.25	T3	3+4	FALSE	2	RP	0	0	Yes
PCa21	Mid. East	59	7.3	T2c	3+4	TRUE	2	RP	-1	0	Yes
PCa22	Mid. East	66	16.2	T2c	3+4	FALSE	2	RP	0	0	Yes
PCa23	Mid. East	58	5.3	T1a	3+3	FALSE	1	RP	0	0	Yes
PCa24	Mid. East	64	5.8	T2c	3+3	FALSE	1	RP	NA	NA	Yes
PCa25	Mid. East	57	9	T2c	3+3	FALSE	1	RP	0	0	Yes
PCa26	Mid. East	63	5.39	T2c	3+4	FALSE	2	RP	0	0	No
PCa27	Mid. East	75	40.35	T3b	4+4	FALSE	4	RP	0	0	No
PCa28	Mid. East	67	62	T4	5+4	FALSE	5	TURP	0	0	No
PCa29	Mid. East	64	6.8	T2c	3+4	FALSE	2	RP	0	0	No
PCa30	Mid. East	65	25	T2c	3+4	FALSE	2	RP	0	0	No
PCa31	Mid. East	75	9.52	T3b	4+3	FALSE	3	RP	0	0	No
PCa32	Mid. East	80	23	T2b	4+4	FALSE	4	RP	0	0	No
PCa33	Mid. East	74	209.4	T3	4+3	TRUE	3	RP	-1	0	No
PCa34	Mid. East	60	40	T3b	3+4	TRUE	2	RP	0	0	No
PCa35	Mid. East	64	2.8	T2a	3+3	FALSE	1	RP	0	0	No
PCa36	Mid. East	67	7	T2c	4+3	FALSE	3	RP	-1	1	No
PCa37	Mid. East	63	5.23	T2c	3+4	FALSE	2	RP	0	0	No
PCa38	Mid. East	68	3.2	T2c	3+4	FALSE	2	RP	-1	0	No
PCa39	Mid. East	66	9.6	T2c	3+4	FALSE	2	RP	0	0	No
PCa40	Mid. East	64	67	T2c	3+4	FALSE	2	RP	0	0	No
PCa41	Mid. East	72	22	T2c	3+4	FALSE	2	RP	0	0	No
PCa42	Mid. East	79	25.5	T3b	3+3	FALSE	1	RP	0	0	No
PCa43	Mid. East	63	24.6	T3b	3+4	FALSE	2	RP	0	0	No
PCa44	Mid. East	66	9	T2c	3+3	FALSE	1	RP	0	0	No
PCa45	Mid. East	79	4.1	T1c	4+5	TRUE	5	RP	0	0	No
PCa46	Mid. East	66	3.35	T2c	3+4	FALSE	2	RP	0	0	No
CPCG0001-F1	Europe	69	7.7	T2b	3+4	FALSE	2	IGRT	NA	NA	Yes
CPCG0002-F1	Africa	65	19	T2a	3+4	TRUE	2	IGRT	NA	NA	Yes
CPCG0003-F1	Europe	72	10	T2a	4+3	TRUE	3	IGRT	NA	NA	Yes
CPCG0004-F1	Europe	76	14.1	T2b	4+3	FALSE	3	IGRT	NA	NA	Yes
CPCG0005-F1	Europe	72	5.4	T2a	3+4	FALSE	2	IGRT	NA	NA	Yes
CPCG0006-F1	Europe	74	8.4	T2b	4+3	FALSE	3	IGRT	NA	NA	Yes
CPCG0007-F1	Africa	66	8.4	T1c	3+4	TRUE	2	IGRT	NA	NA	Yes
CPCG0008-F1	Europe	74	4.7	T2a	3+3	FALSE	1	IGRT	NA	NA	Yes
CPCG0009-F1	Europe	77	8	T2a	3+4	FALSE	2	IGRT	NA	NA	Yes
CPCG0015-F1	Europe	70	5.6	T2a	3+4	TRUE	2	IGRT	NA	NA	Yes
CPCG0019-F1	Europe	71	7.3	T2a	3+4	FALSE	2	IGRT	NA	NA	Yes
CPCG0020-F1	Europe	71	16	T1c	3+3	FALSE	1	IGRT	NA	NA	Yes
CPCG0022-F1	Europe	70	12.6	T1c	3+3	NA	1	IGRT	NA	NA	Yes
CPCG0027-F1	Europe	65	5	T1c	3+3	NA	1	IGRT	NA	NA	Yes
CPCG0030-F1	Europe	73	8	T1c	3+3	FALSE	1	IGRT	NA	NA	Yes
CPCG0040-F1	Europe	71	9	T1c	3+4	FALSE	2	IGRT	NA	NA	Yes
CPCG0042-F1	Europe	76	5.6	T2b	3+4	FALSE	2	IGRT	NA	NA	Yes
CPCG0043-F1	Europe	73	10.2	T2b	3+4	FALSE	2	IGRT	NA	NA	Yes
CPCG0046-F1	Europe	79	5.1	T1c	4+3	FALSE	3	IGRT	NA	NA	Yes
CPCG0047-F1	Europe	72	13.2	T2c	3+4	TRUE	2	IGRT	NA	NA	Yes
CPCG0048-F1	Europe	74	5.1	T2b	3+3	NA	1	IGRT	NA	NA	Yes
CPCG0050-F1	Europe	76	7.7	T1c	4+3	FALSE	3	IGRT	NA	NA	Yes
CPCG0057-F1	Europe	70	10	T2a	3+4	FALSE	2	IGRT	NA	NA	Yes
CPCG0059-F1	Europe	66	11.4	T1c	3+4	FALSE	2	IGRT	NA	NA	Yes
CPCG0063-F1	Europe	69	3.8	T2a	3+4	TRUE	2	IGRT	NA	NA	Yes

CPCG0067-F1	Europe	62	10	T2b	3+4	FALSE		2	IGRT	NA	NA	Yes
CPCG0069-F1	Europe	76	4.3	T2a	3+4	FALSE		2	IGRT	NA	NA	Yes
CPCG0070-F1	Europe	67	5.9	T2a	3+4	FALSE		2	IGRT	NA	NA	Yes
CPCG0071-F1	Europe	75	14.7	T1c	4+3	NA		3	IGRT	NA	NA	Yes
CPCG0072-F1	Europe	76	9.3	T2a	3+4	FALSE		2	IGRT	NA	NA	Yes
CPCG0073-F1	Europe	72	10.3	T1c	3+4	NA		2	IGRT	NA	NA	Yes
CPCG0074-F1	Africa	77	6.3	T2a	3+4	NA		2	IGRT	NA	NA	Yes
CPCG0075-F1	Europe	71	4.1	T2a	3+4	TRUE		2	IGRT	NA	NA	Yes
CPCG0076-F1	Europe	77	12.7	T2a	3+3	FALSE		1	IGRT	NA	NA	Yes
CPCG0078-F1	Europe	61	3.3	T2b	3+4	TRUE		2	IGRT	NA	NA	Yes
CPCG0081-F1	Europe	75	17.8	T2a	4+3	FALSE		3	IGRT	NA	NA	Yes
CPCG0082-F1	Europe	70	7.8	T2a	4+3	TRUE		3	IGRT	NA	NA	Yes
CPCG0083-F1	Europe	61	12.7	T2b	3+4	TRUE		2	IGRT	NA	NA	Yes
CPCG0084-F1	E. Asian	71	7	T2b	3+3	FALSE		1	IGRT	NA	NA	Yes
CPCG0087-F1	Europe	73	9.4	T1c	3+3	FALSE		1	IGRT	NA	NA	Yes
CPCG0089-F1	Europe	74	4.8	T1c	3+3	NA		1	IGRT	NA	NA	Yes
CPCG0090-F1	Europe	78	11.2	T1c	3+3	FALSE		1	IGRT	NA	NA	Yes
CPCG0091-F1	Europe	82	10.4	T1c	4+3	FALSE		3	IGRT	NA	NA	Yes
CPCG0092-F1	Europe	73	11.5	T2a	3+4	NA		2	IGRT	NA	NA	Yes
CPCG0094-F1	Europe	78	16.41	T2b	3+4	FALSE		2	IGRT	NA	NA	Yes
CPCG0095-F1	Europe	69	4	T2a	3+4	FALSE		2	IGRT	NA	NA	Yes
CPCG0096-F1	Africa	71	6.1	T1c	3+4	FALSE		2	IGRT	NA	NA	Yes
CPCG0097-F1	Europe	75	13.7	T1c	3+3	FALSE		1	IGRT	NA	NA	Yes
CPCG0098-F1	Europe	81	7.5	T1c	3+4	TRUE		2	IGRT	NA	NA	Yes
CPCG0099-F1	Europe	66	6.96	T1c	3+4	FALSE		2	RadP	NA	NA	Yes
CPCG0100-F1	Europe	55	5.56	T2a	3+4	TRUE		2	RadP	NA	NA	Yes
CPCG0107-F1	Europe	68	8.1	T1c	3+4	NA		2	IGRT	NA	NA	Yes
CPCG0114-F1	Europe	68	4.4	T2b	3+4	NA		2	IGRT	NA	NA	Yes
CPCG0117-F1	Europe	71	10.1	T2a	3+5	FALSE		4	IGRT	NA	NA	Yes
CPCG0119-F1	Africa	75	7.5	T2a	3+4	FALSE		2	IGRT	NA	NA	Yes
CPCG0120-F1	Europe	73	14.79	T2a	NA	NA	NA		IGRT	NA	NA	Yes
CPCG0121-F1	Europe	64	7.2	T1c	3+4	NA		2	IGRT	NA	NA	Yes
CPCG0122-F1	Europe	66	6.4	T1c	4+3	NA		3	IGRT	NA	NA	Yes
CPCG0123-F1	Europe	71	8.8	T2a	3+4	FALSE		2	IGRT	NA	NA	Yes
CPCG0124-F1	Europe	72	7.2	T2a	3+3	FALSE		1	IGRT	NA	NA	Yes
CPCG0125-F1	Europe	78	7.2	T1c	3+4	FALSE		2	IGRT	NA	NA	Yes
CPCG0127-F1	Native Ameri	54	29.9	T2a	4+3	NA		3	IGRT	NA	NA	Yes
CPCG0128-F1	Africa	70	30.3	T1c	3+3	FALSE		1	IGRT	NA	NA	Yes
CPCG0129-F1	Europe	76	16.7	T1c	3+4	NA		2	IGRT	NA	NA	Yes
CPCG0131-F1	Europe	64	3.4	T1c	3+4	NA		2	IGRT	NA	NA	Yes
CPCG0132-F1	Europe	63	11.4	T2a	3+4	FALSE		2	IGRT	NA	NA	Yes
CPCG0154-F1	Europe	55	13.8	T2a	3+4	FALSE		2	IGRT	NA	NA	Yes
CPCG0158-F1	Europe	70	10	T1c	3+4	FALSE		2	IGRT	NA	NA	Yes
CPCG0166-F1	Europe	74	6.8	T2a	4+3	FALSE		3	IGRT	NA	NA	Yes
CPCG0183-F1	Europe	64	5.67	T1c	3+3	FALSE		1	RadP	NA	NA	Yes
CPCG0184-F1	Europe	52	5.53	T2b	3+4	TRUE		2	RadP	NA	NA	Yes
CPCG0185-F1	Europe	60	4.49	T2a	3+4	FALSE		2	RadP	NA	NA	Yes
CPCG0187-F1	Europe	72	13	T1c	4+3	FALSE		3	IGRT	NA	NA	Yes
CPCG0188-F1	Europe	59	7.23	T1c	3+4	TRUE		2	RadP	NA	NA	Yes
CPCG0189-F1	Europe	57	6.52	T2a	3+4	FALSE		2	RadP	NA	NA	Yes
CPCG0190-F1	Europe	74	4.3	T2b	4+3	FALSE		3	RadP	NA	NA	Yes
CPCG0191-F1	Europe	61	11.1	T2a	3+3	FALSE		1	RadP	NA	NA	Yes
CPCG0192-F1	Europe	52	1.97	T2a	4+5	NA		5	RadP	NA	NA	Yes
CPCG0194-F1	Europe	70	8.7	T1c	3+4	FALSE		2	IGRT	NA	NA	Yes
CPCG0196-F1	Europe	52	4.88	T2b	3+4	TRUE		2	RadP	NA	NA	Yes
CPCG0198-F1	Africa	61	5.2	T2a	3+4	FALSE		2	IGRT	NA	NA	Yes
CPCG0199-F1	Europe	74	6.3	T2a	3+4	NA		2	IGRT	NA	NA	Yes
CPCG0200-F1	Europe	68	6.1	T1c	3+4	FALSE		2	IGRT	NA	NA	Yes
CPCG0201-F1	Europe	63	1.6	T1c	4+3	TRUE		3	IGRT	NA	NA	Yes
CPCG0203-F1	Europe	62	2.7	T2a	3+4	FALSE		2	IGRT	NA	NA	Yes
CPCG0204-F1	Europe	76	9.7	T1b	4+3	FALSE		3	IGRT	NA	NA	Yes
CPCG0205-F1	Europe	65	20.4	T2a	3+4	NA		2	IGRT	NA	NA	Yes
CPCG0206-F1	Europe	67	8	T1c	3+3	FALSE		1	IGRT	NA	NA	Yes
CPCG0208-F1	Europe	60	5.7	T2a	3+4	FALSE		2	RadP	NA	NA	Yes
CPCG0210-F1	Europe	51	3.51	T1c	3+4	FALSE		2	RadP	NA	NA	Yes
CPCG0211-F1	Europe	75	4.5	T2a	3+3	FALSE		1	IGRT	NA	NA	Yes
CPCG0212-F1	Europe	71	7	T1c	3+3	NA		1	IGRT	NA	NA	Yes
CPCG0213-F1	Europe	67	9.3	T2a	4+3	FALSE		3	IGRT	NA	NA	Yes
CPCG0217-F1	Europe	61	6.5	T2a	3+4	FALSE		2	RadP	NA	NA	Yes
CPCG0219-F1	Europe	72	5.3	T2a	3+4	NA		2	IGRT	NA	NA	Yes
CPCG0220-F1	Europe	75	33	T1c	3+3	FALSE		1	IGRT	NA	NA	Yes
CPCG0221-F1	Europe	65	11.8	T2a	3+4	FALSE		2	IGRT	NA	NA	Yes

CPCG0222-F1	Europe	75	7.3	T2a	3+4	FALSE	2	IGRT	NA	NA	Yes
CPCG0223-F1	Europe	75	7.7	T2c	3+3	TRUE	1	IGRT	NA	NA	Yes
CPCG0225-F1	Europe	67	8.6	T2b	3+4	NA	2	IGRT	NA	NA	Yes
CPCG0226-F1	Europe	73	8	T1c	4+3	NA	3	IGRT	NA	NA	Yes
CPCG0227-F1	Europe	76	14	T2a	3+3	FALSE	1	IGRT	NA	NA	Yes
CPCG0228-F1	Europe	70	8.1	T2a	3+3	TRUE	1	IGRT	NA	NA	Yes
CPCG0229-F1	Europe	76	11.9	T2a	3+4	FALSE	2	IGRT	NA	NA	Yes
CPCG0232-F1	Africa	72	8.15	T1c	3+4	FALSE	2	RadP	NA	NA	Yes
CPCG0233-F1	Africa	70	4.63	T1c	4+3	FALSE	3	RadP	NA	NA	Yes
CPCG0234-F1	E. Asian	70	5.7	T1c	3+3	FALSE	1	IGRT	NA	NA	Yes
CPCG0235-F1	Africa	59	8.04	T2a	4+3	TRUE	3	RadP	NA	NA	Yes
CPCG0236-F1	Europe	59	10.92	T2a	4+3	TRUE	3	RadP	NA	NA	Yes
CPCG0237-F1	Europe	60	10.7	T2a	3+3	FALSE	1	IGRT	NA	NA	Yes
CPCG0238-F1	Europe	56	7.92	T2b	3+4	TRUE	2	RadP	NA	NA	Yes
CPCG0240-F1	Europe	65	2.7	T2a	3+3	FALSE	1	IGRT	NA	NA	Yes
CPCG0241-F1	Europe	67	5.64	T1c	3+4	TRUE	2	RadP	NA	NA	Yes
CPCG0242-F1	Europe	56	4	T2a	3+4	TRUE	2	RadP	NA	NA	Yes
CPCG0243-F1	Europe	64	13.6	T2b	3+3	FALSE	1	IGRT	NA	NA	Yes
CPCG0246-F1	E. Asian	61	5.64	T1c	3+4	FALSE	2	RadP	NA	NA	Yes
CPCG0248-F1	Europe	54	5.1	T2a	3+4	TRUE	2	RadP	NA	NA	Yes
CPCG0249-F1	E. Asian	66	7.1	T1c	3+4	FALSE	2	RadP	NA	NA	Yes
CPCG0250-F1	E. Asian	62	4.23	T2b	3+4	FALSE	2	RadP	NA	NA	Yes
CPCG0251-F1	Europe	52	13.41	T2a	3+4	FALSE	2	RadP	NA	NA	Yes
CPCG0255-F1	Europe	72	6.9	T1c	3+4	FALSE	2	RadP	NA	NA	Yes
CPCG0256-F1	Europe	64	7.32	T1c	3+4	FALSE	2	RadP	NA	NA	Yes
CPCG0257-F1	Europe	75	8	T2a	3+3	NA	1	IGRT	NA	NA	Yes
CPCG0258-F1	Europe	49	5.13	T1c	3+4	FALSE	2	RadP	NA	NA	Yes
CPCG0259-F1	Europe	56	11.65	T1c	3+4	FALSE	2	RadP	NA	NA	Yes
CPCG0260-F1	Europe	65	6.59	T2a	3+4	TRUE	2	RadP	NA	NA	Yes
CPCG0262-F1	Europe	63	5.7	T2a	3+4	FALSE	2	RadP	NA	NA	Yes
CPCG0263-F1	Africa	63	13.9	T2a	3+4	FALSE	2	RadP	NA	NA	Yes
CPCG0265-F1	Europe	63	9.34	T1c	3+4	FALSE	2	RadP	NA	NA	Yes
CPCG0266-F1	Europe	66	1.74	T2a	3+4	FALSE	2	RadP	NA	NA	Yes
CPCG0267-F1	Africa	45	17.1	T2a	4+3	FALSE	3	RadP	NA	NA	Yes
CPCG0268-F1	Europe	59	12.35	T2b	3+4	FALSE	2	RadP	NA	NA	Yes
CPCG0269-F1	Europe	68	13.21	T1c	3+3	FALSE	1	RadP	NA	NA	Yes
CPCG0271-F1	E. Asian	63	7.81	T2a	4+3	FALSE	3	RadP	NA	NA	Yes
CPCG0274-F1	E. Asian	81	2.1	T2a	3+4	NA	2	IGRT	NA	NA	Yes
CPCG0277-F1	Europe	78	4.9	T1c	4+3	FALSE	3	IGRT	NA	NA	Yes
CPCG0278-F1	Europe	73	6.42	T2a	4+4	TRUE	4	IGRT	NA	NA	Yes
CPCG0279-F1	Europe	72	14	T2c	3+4	FALSE	2	IGRT	NA	NA	Yes
CPCG0280-F1	Europe	75	5	T1c	3+4	FALSE	2	IGRT	NA	NA	Yes
CPCG0281-F1	Europe	83	10.5	T1c	3+4	FALSE	2	IGRT	NA	NA	Yes
CPCG0282-F1	Europe	69	8.2	T2a	3+4	TRUE	2	IGRT	NA	NA	Yes
CPCG0283-F1	Europe	58	4.7	T2a	4+3	FALSE	3	IGRT	NA	NA	Yes
CPCG0285-F1	Europe	77	9.44	T1c	3+4	FALSE	2	RadP	NA	NA	Yes
CPCG0286-F1	Europe	72	9	T1c	3+4	FALSE	2	IGRT	NA	NA	Yes
CPCG0287-F1	Europe	76	8.5	T2a	3+5	FALSE	4	IGRT	NA	NA	Yes
CPCG0288-F1	Europe	74	12.9	T2a	4+3	FALSE	3	IGRT	NA	NA	Yes
CPCG0289-F1	E. Asian	71	4.4	T2a	4+3	NA	3	IGRT	NA	NA	Yes
CPCG0290-F1	Europe	75	18	T2c	4+4	TRUE	4	IGRT	NA	NA	Yes
CPCG0291-F1	Europe	75	8.2	T2b	3+4	FALSE	2	IGRT	NA	NA	Yes
CPCG0292-F1	Europe	70	7.8	T2b	3+3	FALSE	1	IGRT	NA	NA	Yes
CPCG0293-F1	Europe	64	6.2	T2a	NA	NA	NA	IGRT	NA	NA	Yes
CPCG0294-F1	Europe	78	5.7	T2a	3+4	NA	2	IGRT	NA	NA	Yes
CPCG0295-F1	Europe	77	12.4	T2a	3+4	FALSE	2	IGRT	NA	NA	Yes
CPCG0296-F1	Europe	61	10.5	T2a	3+4	TRUE	2	IGRT	NA	NA	Yes
CPCG0297-F1	Europe	65	2.2	T2b	3+4	NA	2	IGRT	NA	NA	Yes
CPCG0298-F1	Europe	65	15.15	T2a	3+5	FALSE	4	IGRT	NA	NA	Yes
CPCG0299-F1	Europe	69	17.3	T1c	4+3	TRUE	3	IGRT	NA	NA	Yes
CPCG0300-F1	Europe	68	5.35	T1c	3+4	NA	2	IGRT	NA	NA	Yes
CPCG0301-F1	Europe	74	7.8	T2b	3+4	FALSE	2	IGRT	NA	NA	Yes
CPCG0302-F1	Europe	75	6	T2b	3+4	FALSE	2	IGRT	NA	NA	Yes
CPCG0303-F1	Europe	78	22	T1c	3+4	FALSE	2	IGRT	NA	NA	Yes
CPCG0304-F1	Europe	67	8.9	T2a	3+3	FALSE	1	IGRT	NA	NA	Yes
CPCG0305-F1	Europe	68	5	T1c	3+5	FALSE	4	IGRT	NA	NA	Yes
CPCG0306-F1	Europe	69	3.8	T2b	4+3	FALSE	3	IGRT	NA	NA	Yes
CPCG0307-F1	Europe	59	10.6	T1c	4+3	TRUE	3	IGRT	NA	NA	Yes
CPCG0308-F1	E. Asian	71	3.5	T1c	3+3	FALSE	1	IGRT	NA	NA	Yes
CPCG0309-F1	Europe	72	10.7	T1c	3+3	NA	1	IGRT	NA	NA	Yes
CPCG0310-F1	Europe	57	10.08	T1c	4+5	FALSE	5	IGRT	NA	NA	Yes
CPCG0311-F1	Europe	70	4.2	T1c	3+3	FALSE	1	IGRT	NA	NA	Yes

CPCG0312-F1	Europe	74	13.1	T1c	3+3	FALSE	1	IGRT	NA	NA	Yes
CPCG0313-F1	Europe	71	5.8	T2a	4+3	TRUE	3	IGRT	NA	NA	Yes
CPCG0314-F1	Europe	65	8.03	T1c	3+4	FALSE	2	IGRT	NA	NA	Yes
CPCG0315-F1	Europe	70	8.6	T2a	4+3	TRUE	3	IGRT	NA	NA	Yes
CPCG0316-F1	Europe	79	7.7	T2a	3+3	TRUE	1	IGRT	NA	NA	Yes
CPCG0317-F1	Europe	66	13	T2c	3+4	TRUE	2	IGRT	NA	NA	Yes
CPCG0318-F1	Europe	70	6.3	T1c	3+3	TRUE	1	IGRT	NA	NA	Yes
CPCG0319-F1	Europe	80	6.9	T1c	3+3	FALSE	1	IGRT	NA	NA	Yes
CPCG0320-F1	Europe	74	10.64	T2b	3+4	NA	2	IGRT	NA	NA	Yes
CPCG0321-F1	Europe	72	7.8	T1b	3+4	FALSE	2	IGRT	NA	NA	Yes
CPCG0322-F1	Europe	74	10.7	T1c	4+3	FALSE	3	IGRT	NA	NA	Yes
CPCG0323-F1	Europe	75	13.6	T2b	4+3	FALSE	3	IGRT	NA	NA	Yes
CPCG0324-F1	Europe	65	12.1	T2a	3+4	TRUE	2	RadP	NA	NA	Yes
CPCG0326-F1	Europe	75	0.72	T1c	3+3	NA	1	IGRT	NA	NA	Yes
CPCG0327-F1	Africa	57	5.1	T1c	4+3	FALSE	3	IGRT	NA	NA	Yes
CPCG0328-F1	Europe	66	15.46	T2a	3+3	NA	1	IGRT	NA	NA	Yes
CPCG0329-F1	Europe	73	7.35	T2a	4+3	FALSE	3	IGRT	NA	NA	Yes
CPCG0330-F1	Europe	71	6.9	T2a	3+4	TRUE	2	IGRT	NA	NA	Yes
CPCG0331-F1	Europe	70	11.08	T2a	3+4	TRUE	2	RadP	NA	NA	Yes
CPCG0333-F1	Europe	61	9.79	T1c	4+3	FALSE	3	RadP	NA	NA	Yes
CPCG0334-F1	Europe	54	7.41	T2a	3+4	TRUE	2	RadP	NA	NA	Yes
CPCG0335-F1	Europe	63	14.06	T1c	4+3	TRUE	3	RadP	NA	NA	Yes
CPCG0336-F1	Europe	61	9.08	T1c	3+4	FALSE	2	RadP	NA	NA	Yes
CPCG0339-F1	Native Ameri	58	14.29	T1c	3+4	FALSE	2	RadP	NA	NA	Yes
CPCG0340-F1	Europe	62	4.41	T1c	3+4	FALSE	2	RadP	NA	NA	Yes
CPCG0341-F1	Europe	57	11.3	T1c	3+4	FALSE	2	RadP	NA	NA	Yes
CPCG0342-F1	Europe	52	6.3	T2a	4+3	FALSE	3	RadP	NA	NA	Yes
CPCG0344-F1	Europe	61	5.5	T1c	3+4	FALSE	2	RadP	NA	NA	Yes
CPCG0345-F1	E. Asian	60	6.6	T2a	3+4	FALSE	2	RadP	NA	NA	Yes
CPCG0346-F1	Europe	60	6.5	T1c	3+4	TRUE	2	RadP	NA	NA	Yes
CPCG0347-F1	Europe	75	6.7	T2b	3+4	NA	2	IGRT	NA	NA	Yes
CPCG0348-F1	Europe	57	4.22	T1c	3+4	FALSE	2	RadP	NA	NA	Yes
CPCG0349-F1	Europe	62	7.1	T1c	3+4	TRUE	2	RadP	NA	NA	Yes
CPCG0350-F1	Europe	53	39.47	T1c	3+3	TRUE	1	RadP	NA	NA	Yes
CPCG0352-F1	Europe	56	9.1	T1c	3+3	TRUE	1	RadP	NA	NA	Yes
CPCG0353-F1	Europe	66	9	T1c	4+3	FALSE	3	RadP	NA	NA	Yes
CPCG0354-F1	Europe	61	6.7	T1c	3+4	FALSE	2	RadP	NA	NA	Yes
CPCG0355-F1	Europe	55	6.7	T1c	3+3	FALSE	1	RadP	NA	NA	Yes
CPCG0356-F1	Europe	52	8.49	T2b	3+4	TRUE	2	RadP	NA	NA	Yes
CPCG0357-F1	Europe	59	3.11	T2b	3+4	TRUE	2	RadP	NA	NA	Yes
CPCG0358-F1	Europe	69	5.88	T1c	3+3	FALSE	1	RadP	NA	NA	Yes
CPCG0360-F1	Europe	66	14	T2b	3+4	TRUE	2	RadP	NA	NA	Yes
CPCG0361-F1	Europe	70	4.6	T2a	3+4	TRUE	2	RadP	NA	NA	Yes
CPCG0362-F1	Europe	58	8.77	T2a	4+3	FALSE	3	RadP	NA	NA	Yes
CPCG0363-F1	Europe	57	4.27	T1c	3+4	FALSE	2	RadP	NA	NA	Yes
CPCG0364-F1	Europe	63	8.11	T2b	3+4	TRUE	2	RadP	NA	NA	Yes
CPCG0365-F1	Europe	72	4.23	T1c	3+4	FALSE	2	RadP	NA	NA	Yes
CPCG0366-F1	Europe	61	15	T2c	3+4	FALSE	2	RadP	NA	NA	Yes
CPCG0368-F1	Europe	64	4.39	T1c	3+4	FALSE	2	RadP	NA	NA	Yes
CPCG0369-F1	Europe	64	7.18	T1c	3+3	FALSE	1	RadP	NA	NA	Yes
CPCG0371-F1	Europe	61	7.52	T2a	4+3	TRUE	3	RadP	NA	NA	Yes
CPCG0372-F1	Europe	60	4	T1c	4+3	FALSE	3	RadP	NA	NA	Yes
CPCG0373-F1	Europe	60	4.9	T1c	3+3	TRUE	1	RadP	NA	NA	Yes
CPCG0374-F1	Europe	64	4.7	T1c	3+4	FALSE	2	RadP	NA	NA	Yes
CPCG0375-F1	Europe	70	7.01	T1c	3+3	FALSE	1	RadP	NA	NA	Yes
CPCG0377-F1	Europe	77	6.9	T1c	4+3	FALSE	3	RadP	NA	NA	Yes
CPCG0378-F1	Europe	69	8.7	T2a	3+4	TRUE	2	RadP	NA	NA	Yes
CPCG0379-F1	Europe	69	14.6	T2b	3+4	TRUE	2	RadP	NA	NA	Yes
CPCG0380-F1	Europe	58	6.29	T1c	3+4	TRUE	2	RadP	NA	NA	Yes
CPCG0381-F1	Europe	65	5.76	T1c	3+4	FALSE	2	RadP	NA	NA	Yes
CPCG0382-F1	Europe	71	6.8	T1c	3+4	TRUE	2	RadP	NA	NA	Yes
CPCG0387-F1	Europe	67	4.52	T2b	4+3	FALSE	3	RadP	NA	NA	Yes
CPCG0388-F1	Europe	54	2.5	T2a	3+4	FALSE	2	RadP	NA	NA	Yes
CPCG0389-F1	Europe	72	9.3	T2b	3+4	NA	2	RadP	NA	NA	Yes
CPCG0390-F1	Europe	75	6.23	T1c	4+3	NA	3	RadP	NA	NA	Yes
CPCG0391-F1	Europe	61	5.3	T1c	3+4	NA	2	RadP	NA	NA	Yes
CPCG0392-F1	Europe	61	19.5	T2a	3+4	TRUE	2	RadP	NA	NA	Yes
CPCG0394-F1	Europe	51	2.5	T1c	3+4	FALSE	2	RadP	NA	NA	Yes
CPCG0395-F1	Europe	68	10.16	T2a	3+4	TRUE	2	RadP	NA	NA	Yes
CPCG0396-F1	Europe	59	17.98	T1c	4+3	FALSE	3	RadP	NA	NA	Yes
CPCG0397-F1	Europe	66	8.21	T2a	4+3	TRUE	3	RadP	NA	NA	Yes
CPCG0398-F1	Europe	69	3.35	T2a	3+4	TRUE	2	RadP	NA	NA	Yes

CPCG0401-F1	Europe	58	3.6	T1c	3+4	TRUE	2	RadP	NA	NA	Yes
CPCG0402-F1	Europe	61	4.48	T2c	4+3	TRUE	3	RadP	NA	NA	Yes
CPCG0404-F1	Europe	56	3.6	T1c	3+4	FALSE	2	RadP	NA	NA	Yes
CPCG0406-F1	Europe	55	4.84	T1c	3+4	FALSE	2	RadP	NA	NA	Yes
CPCG0407-F1	Europe	58	11.5	T1c	3+3	FALSE	1	RadP	NA	NA	Yes
CPCG0408-F1	Europe	68	3.47	T1a	4+4	TRUE	4	RadP	NA	NA	Yes
CPCG0409-F1	Europe	59	6.3	T1c	3+4	FALSE	2	RadP	NA	NA	Yes
CPCG0410-F1	Europe	62	5	T2b	3+4	TRUE	2	RadP	NA	NA	Yes
CPCG0411-F1	Europe	46	10.55	T1c	3+4	FALSE	2	RadP	NA	NA	Yes
CPCG0412-F1	Europe	59	3.36	T2b	4+3	TRUE	3	RadP	NA	NA	Yes
CPCG0413-F1	Europe	59	6.89	T2a	3+4	FALSE	2	RadP	NA	NA	Yes
CPCG0414-F1	Native Ameri	44	5.4	T1b	3+3	FALSE	1	RadP	NA	NA	Yes
CPCG0415-F1	Europe	57	5.5	T1c	3+4	FALSE	2	RadP	NA	NA	Yes
CPCG0416-F1	Europe	60	4.8	T2b	3+4	FALSE	2	RadP	NA	NA	Yes
CPCG0418-F1	Europe	67	4.32	T1c	3+4	FALSE	2	RadP	NA	NA	Yes
CPCG0423-F1	Europe	62	3.4	T1c	3+3	FALSE	1	RadP	NA	NA	Yes
CPCG0424-F1	Europe	59	10	T2a	3+3	FALSE	1	RadP	NA	NA	Yes
CPCG0426-F1	Europe	59	4.8	T1c	4+3	TRUE	3	RadP	NA	NA	Yes
CPCG0428-F1	Europe	59	7	T1c	4+3	FALSE	3	RadP	NA	NA	Yes
CPCG0432-F1	Europe	56	13.3	T2b	3+4	NA	2	RadP	NA	NA	Yes
CPCG0433-F1	Europe	59	6.6	T2b	3+3	NA	1	RadP	NA	NA	Yes
CPCG0434-F1	Europe	63	8.3	T1c	3+4	FALSE	2	RadP	NA	NA	Yes
CPCG0435-F1	Europe	52	10.83	T2a	4+3	FALSE	3	RadP	NA	NA	Yes
CPCG0437-F1	Europe	68	6.9	T2a	3+4	TRUE	2	RadP	NA	NA	Yes
CPCG0439-F1	Europe	73	4.23	T2a	3+4	TRUE	2	RadP	NA	NA	Yes
CPCG0445-F1	Europe	61	3.6	T2a	4+3	TRUE	3	RadP	NA	NA	Yes
CPCG0448-F1	Europe	47	11.19	T1c	3+4	TRUE	2	RadP	NA	NA	Yes
CPCG0450-F1	Europe	63	3.03	T1c	3+4	FALSE	2	NA	NA	NA	Yes
CPCG0451-F1	Europe	62	7.9	T2b	3+4	FALSE	2	RadP	NA	NA	Yes
CPCG0452-F1	Europe	56	9.1	T1c	3+3	FALSE	1	RadP	NA	NA	Yes
CPCG0453-F1	Europe	63	10	T2a	3+4	TRUE	2	RadP	NA	NA	Yes
CPCG0454-F1	Europe	53	9.2	T2a	3+3	FALSE	1	RadP	NA	NA	Yes
CPCG0455-F1	Europe	62	4.85	T1c	3+4	FALSE	2	RadP	NA	NA	Yes
CPCG0456-F1	Europe	65	6	T2b	3+4	FALSE	2	RadP	NA	NA	Yes
CPCG0457-F1	Europe	66	4.92	T2b	3+4	TRUE	2	RadP	NA	NA	Yes
CPCG0458-F1	Europe	52	15.58	T1c	3+4	NA	2	RadP	NA	NA	Yes
CPCG0459-F1	Europe	67	9.42	T1c	3+4	NA	2	RadP	NA	NA	Yes
CPCG0462-F1	Europe	63	11.65	T2b	4+3	NA	3	RadP	NA	NA	Yes
CPCG0463-F1	Europe	69	11.2	T1c	3+3	NA	1	RadP	NA	NA	Yes
CPCG0464-F1	Europe	57	4.46	T2a	3+4	NA	2	RadP	NA	NA	Yes
CPCG0465-F1	Europe	50	4.07	T2a	3+4	NA	2	RadP	NA	NA	Yes
CPCG0466-F1	Europe	55	5.71	T1c	4+3	FALSE	3	RadP	NA	NA	Yes
CPCG0467-F1	Europe	66	6.7	T1c	3+4	FALSE	2	RadP	NA	NA	Yes
CPCG0468-F1	Europe	62	7.7	T1c	4+3	FALSE	3	RadP	NA	NA	Yes
CPCG0470-F1	Europe	60	3.1	T1c	4+3	FALSE	3	RadP	NA	NA	Yes
CPCG0471-F1	Europe	73	7.6	T2a	4+3	TRUE	3	RadP	NA	NA	Yes
CPCG0472-F1	Europe	46	10.66	T2b	3+3	TRUE	1	RadP	NA	NA	Yes
CPCG0474-F1	Europe	57	4.6	T1c	4+3	FALSE	3	RadP	NA	NA	Yes
CPCG0482-F1	Europe	62	5	T2b	4+3	FALSE	3	RadP	NA	NA	Yes
CPCG0483-F1	Europe	42	6.2	T1c	4+3	FALSE	3	RadP	NA	NA	Yes
CPCG0484-F1	Europe	62	5.58	T2a	3+4	TRUE	2	RadP	NA	NA	Yes
CPCG0486-F1	Europe	74	7.66	T1c	3+4	FALSE	2	RadP	NA	NA	Yes
CPCG0487-F1	Europe	68	5.3	T1c	3+4	TRUE	2	RadP	NA	NA	Yes
CPCG0489-F1	Europe	67	5.4	T1c	4+3	TRUE	3	RadP	NA	NA	Yes
CPCG0490-F1	Europe	63	13	T1c	3+4	FALSE	2	RadP	NA	NA	Yes
CPCG0492-F1	Europe	59	5	T2a	3+4	FALSE	2	RadP	NA	NA	Yes
CPCG0493-F1	Europe	61	5.6	T1c	4+3	TRUE	3	RadP	NA	NA	Yes
CPCG0496-F1	Native Ameri	73	9.9	T1c	3+4	FALSE	2	RadP	NA	NA	Yes
CPCG0498-F1	Europe	64	5.7	T1c	3+4	FALSE	2	RadP	NA	NA	Yes
CPCG0499-F1	Europe	52	14	T2a	4+3	TRUE	3	RadP	NA	NA	Yes
CPCG0500-F1	Europe	66	6.4	T1c	3+4	FALSE	2	RadP	NA	NA	Yes
CPCG0502-F1	Europe	65	3.8	T2a	3+4	TRUE	2	RadP	NA	NA	Yes
CPCG0503-F1	Europe	65	2	T2b	3+4	FALSE	2	RadP	NA	NA	Yes
CPCG0504-F1	Europe	64	6.2	T1c	3+4	TRUE	2	RadP	NA	NA	Yes
CPCG0507-F1	Europe	64	5.1	T1c	4+3	FALSE	3	RadP	NA	NA	Yes
CPCG0508-F1	Europe	63	10.2	T1c	3+3	FALSE	1	RadP	NA	NA	Yes
CPCG0509-F1	Europe	74	5.9	T2b	4+3	FALSE	3	RadP	NA	NA	Yes
CPCG0514-F1	Europe	66	13.2	T2a	3+4	NA	2	RadP	NA	NA	Yes
CPCG0519-F1	Europe	56	6	T2b	3+3	FALSE	1	RadP	NA	NA	Yes
CPCG0520-F1	Europe	62	8.23	T1c	3+4	FALSE	2	RadP	NA	NA	Yes
CPCG0521-F1	Europe	55	10.1	T2b	3+3	FALSE	1	RadP	NA	NA	Yes
CPCG0522-F1	Europe	59	5.9	T2b	3+4	FALSE	2	RadP	NA	NA	Yes

CPCG0523-F1	Europe	59	3.4	T1c	3+4	FALSE	2	RadP	NA	NA	Yes
CPCG0524-F1	Europe	69	4.8	T1c	3+4	FALSE	2	RadP	NA	NA	Yes
CPCG0525-F1	Europe	69	2	T1c	4+3	FALSE	3	RadP	NA	NA	Yes
CPCG0526-F1	Europe	59	4.5	T2a	3+4	TRUE	2	RadP	NA	NA	Yes
CPCG0527-F1	Europe	62	9.07	T1c	4+3	FALSE	3	RadP	NA	NA	Yes
CPCG0528-F1	Europe	60	10.7	T1c	3+3	TRUE	1	RadP	NA	NA	Yes
CPCG0529-F1	Europe	55	5.8	T1c	3+4	FALSE	2	RadP	NA	NA	Yes
CPCG0531-F1	Europe	69	18	T1c	4+3	FALSE	3	RadP	NA	NA	Yes
CPCG0532-F1	Africa	71	7.9	T1c	3+4	FALSE	2	RadP	NA	NA	Yes
CPCG0534-F1	Europe	62	18	T1c	3+3	FALSE	1	RadP	NA	NA	Yes
CPCG0537-F1	Europe	72	8.3	T2c	4+3	TRUE	3	RadP	NA	NA	Yes
CPCG0539-F1	Europe	59	3.42	T1c	4+3	FALSE	3	RadP	NA	NA	Yes
CPCG0540-F1	Europe	73	7.93	T1c	3+4	NA	2	RadP	NA	NA	Yes
CPCG0545-F1	Europe	67	7.6	T1c	4+3	NA	3	RadP	NA	NA	Yes
CPCG0547-F1	Europe	67	6.84	T1c	3+4	NA	2	RadP	NA	NA	Yes
CPCG0550-F1	Europe	54	6.59	T2a	3+4	NA	2	RadP	NA	NA	Yes
CPCG0551-F1	Europe	67	5.2	T1c	3+4	NA	2	RadP	NA	NA	Yes
CPCG0557-F1	Europe	46	4.74	T2c	3+4	NA	2	RadP	NA	NA	Yes
CPCG0559-F1	Europe	67	4.2	T1c	3+4	FALSE	2	RadP	NA	NA	Yes
CPCG0560-F1	Europe	72	7.6	T1c	3+4	FALSE	2	RadP	NA	NA	Yes
CPCG0561-F1	Europe	56	5.3	T2b	3+4	TRUE	2	RadP	NA	NA	Yes
CPCG0562-F1	Europe	67	7.7	T2b	3+4	FALSE	2	RadP	NA	NA	Yes
CPCG0563-F1	Europe	70	9.6	T2b	3+4	FALSE	2	RadP	NA	NA	Yes
CPCG0565-F1	Europe	72	3.6	T2b	4+3	TRUE	3	RadP	NA	NA	Yes
CPCG0566-F1	Europe	64	10.6	T1c	4+3	TRUE	3	RadP	NA	NA	Yes
CPCG0567-F1	Europe	55	6.6	T1c	3+4	FALSE	2	RadP	NA	NA	Yes
CPCG0568-F1	Europe	66	5.9	T1c	3+4	FALSE	2	RadP	NA	NA	Yes
CPCG0569-F1	Europe	68	5.2	T2b	4+3	FALSE	3	RadP	NA	NA	Yes
CPCG0570-F1	Europe	63	2.1	T2b	3+4	TRUE	2	RadP	NA	NA	Yes
CPCG0571-F1	Europe	69	10	T1c	3+4	TRUE	2	RadP	NA	NA	Yes
CPCG0573-F1	Europe	56	3.8	T2a	3+4	FALSE	2	RadP	NA	NA	Yes
CPCG0574-F1	Europe	62	15	T2a	3+4	TRUE	2	RadP	NA	NA	Yes
CPCG0575-F1	Europe	58	6.4	T1c	3+3	FALSE	1	RadP	NA	NA	Yes
CPCG0576-F1	Europe	56	4.6	T2a	3+4	TRUE	2	RadP	NA	NA	Yes
CPCG0577-F1	Europe	70	13	T1c	3+4	TRUE	2	RadP	NA	NA	Yes
CPCG0578-F1	Europe	64	8.5	T1c	3+4	TRUE	2	RadP	NA	NA	Yes
CPCG0579-F1	Europe	65	8.8	T2a	3+4	TRUE	2	RadP	NA	NA	Yes
CPCG0580-F1	Europe	61	3.2	T1c	3+4	FALSE	2	RadP	NA	NA	Yes
CPCG0581-F1	Europe	65	12.5	T1c	3+3	TRUE	1	RadP	NA	NA	Yes
CPCG0582-F1	Europe	70	13.2	T1c	3+4	FALSE	2	RadP	NA	NA	Yes
CPCG0584-F1	E. Asian	59	5.6	T2a	3+4	FALSE	2	RadP	NA	NA	Yes
CPCG0587-F1	Europe	71	9.4	T2b	4+3	FALSE	3	RadP	NA	NA	Yes
CPCG0588-F1	Europe	61	6.3	T2a	3+4	NA	2	RadP	NA	NA	Yes
CPCG0589-F1	Europe	70	12.2	T1c	3+4	TRUE	2	RadP	NA	NA	Yes
CPCG0590-F1	Europe	66	4.9	T1c	3+4	FALSE	2	RadP	NA	NA	Yes
CPCG0591-F1	Europe	61	6.1	T2c	3+4	TRUE	2	RadP	NA	NA	Yes
CPCG0592-F1	Europe	66	8.6	T2b	3+4	FALSE	2	RadP	NA	NA	Yes
CPCG0593-F1	Europe	65	15	T1c	3+3	FALSE	1	RadP	NA	NA	Yes
CPCG0594-F1	Europe	51	8.6	T1c	3+4	FALSE	2	RadP	NA	NA	Yes
CPCG0595-F1	Europe	54	4.7	T1c	4+3	FALSE	3	RadP	NA	NA	Yes
CPCG0596-F1	Europe	62	7.4	T1c	3+4	TRUE	2	RadP	NA	NA	Yes
CPCG0597-F1	Europe	68	7.4	T2b	3+4	TRUE	2	RadP	NA	NA	Yes
CPCG0598-F1	Europe	63	4.7	T1c	4+3	TRUE	3	RadP	NA	NA	Yes
CPCG0599-F1	Europe	68	10.5	T2a	3+4	FALSE	2	RadP	NA	NA	Yes
CPCG0600-F1	Native Ameri	56	9.4	T1c	3+4	FALSE	2	RadP	NA	NA	Yes
CPCG0601-F1	Europe	58	8.9	T2b	4+3	TRUE	3	RadP	NA	NA	Yes
CPCG0602-F1	Europe	51	6.3	T2a	4+3	FALSE	3	RadP	NA	NA	Yes
CPCG0603-F1	Europe	63	3.8	T2b	3+4	FALSE	2	RadP	NA	NA	Yes
CPCG0604-F1	Europe	60	6.5	T1c	3+4	FALSE	2	RadP	NA	NA	Yes
CPCG0605-F1	Europe	60	4.7	T1c	3+3	FALSE	1	RadP	NA	NA	Yes
CPCG0606-F1	Europe	59	18.7	T2b	3+4	TRUE	2	RadP	NA	NA	Yes
CPCG0607-F1	Europe	74	5.7	T2b	4+3	TRUE	3	RadP	NA	NA	Yes
CPCG0609-F1	Europe	60	4.93	T2a	3+4	FALSE	2	RadP	NA	NA	Yes
