# Supplementary figures and images for: Copy Number Profiles of Prostate Cancer in Men of Middle Eastern Ancestry
Source: Cancers (Basel). 2021 May 14;13(10):2363. doi: 10.3390/cancers13102363 (PMC8153627; doi:10.3390/cancers13102363)

# Supplementary Figure 1

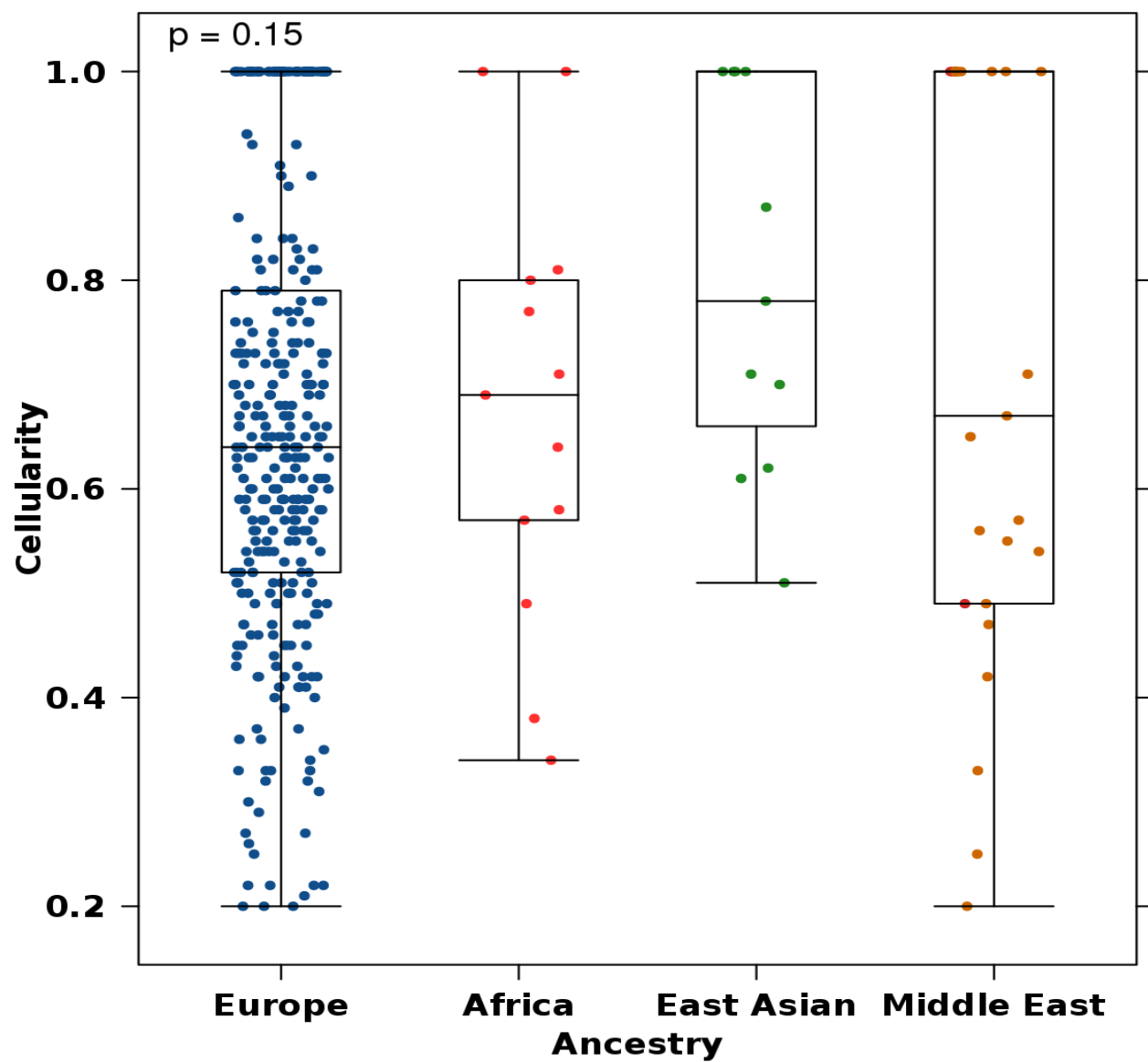

Supplement: Supplementary file 1 [file cancers-13-02363-s001.zip › cancers-1141750 - supple.- revised version/FigureS2.pdf]

## Supplementary Figure 2

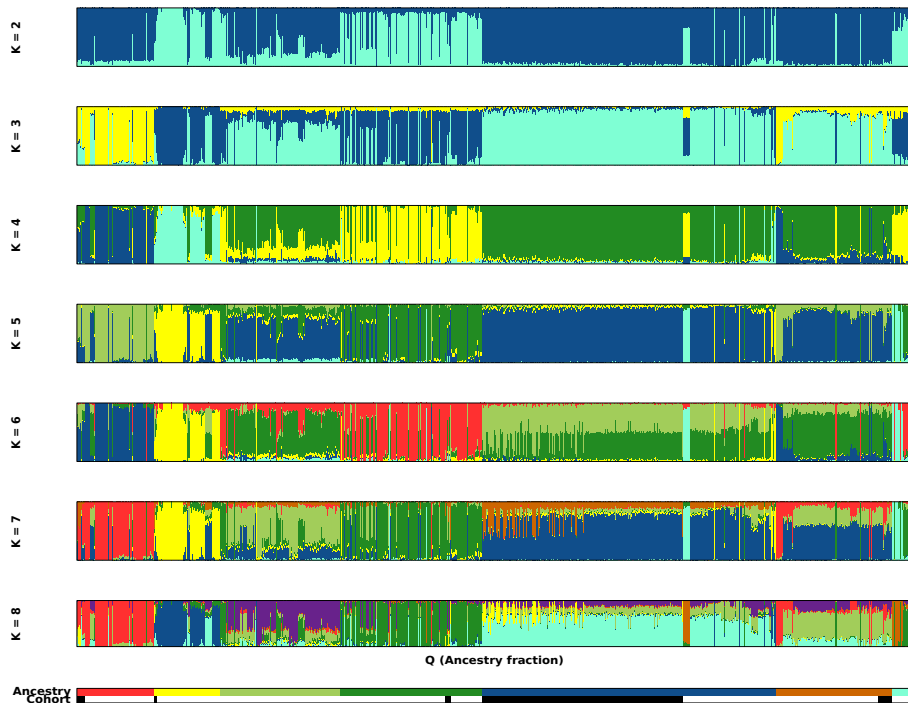

Supplement: Supplementary file 1 [file cancers-13-02363-s001.zip › cancers-1141750 - supple.- revised version/FigureS3.pdf]

# Supplementary Figure 3

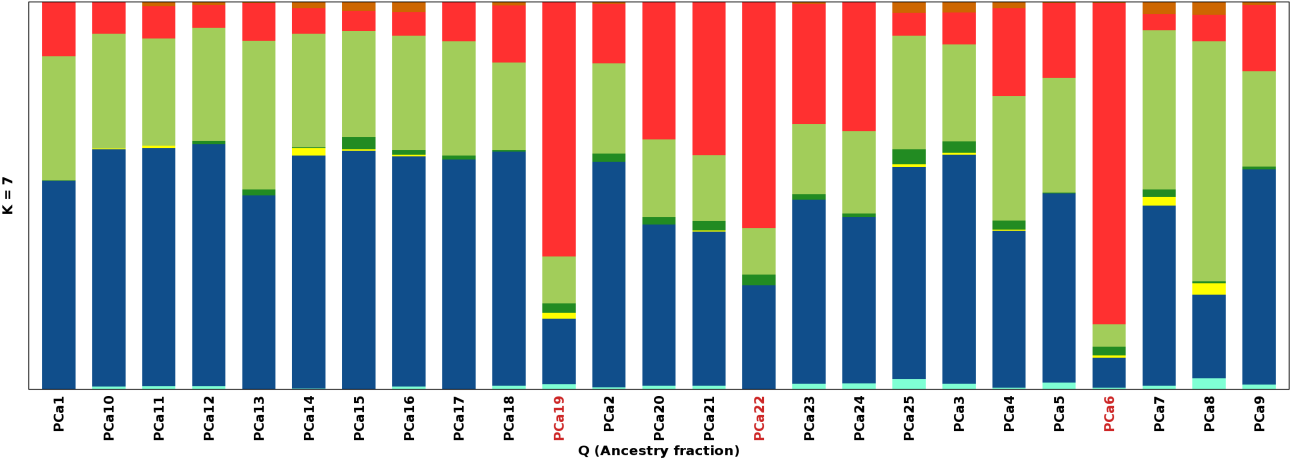

Supplement: Supplementary file 1 [file cancers-13-02363-s001.zip › cancers-1141750 - supple.- revised version/FigureS4.pdf]

# Supplementary Figure 4

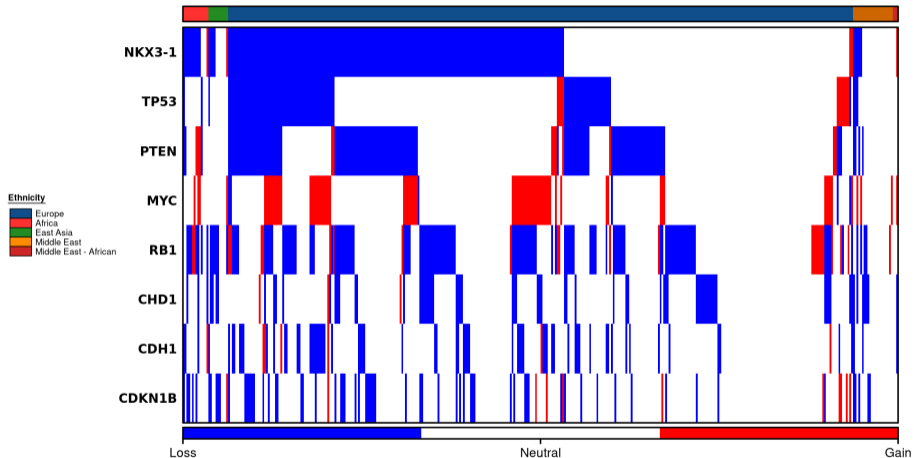

Supplement: Supplementary file 1 [file cancers-13-02363-s001.zip › cancers-1141750 - supple.- revised version/FigureS5.pdf]

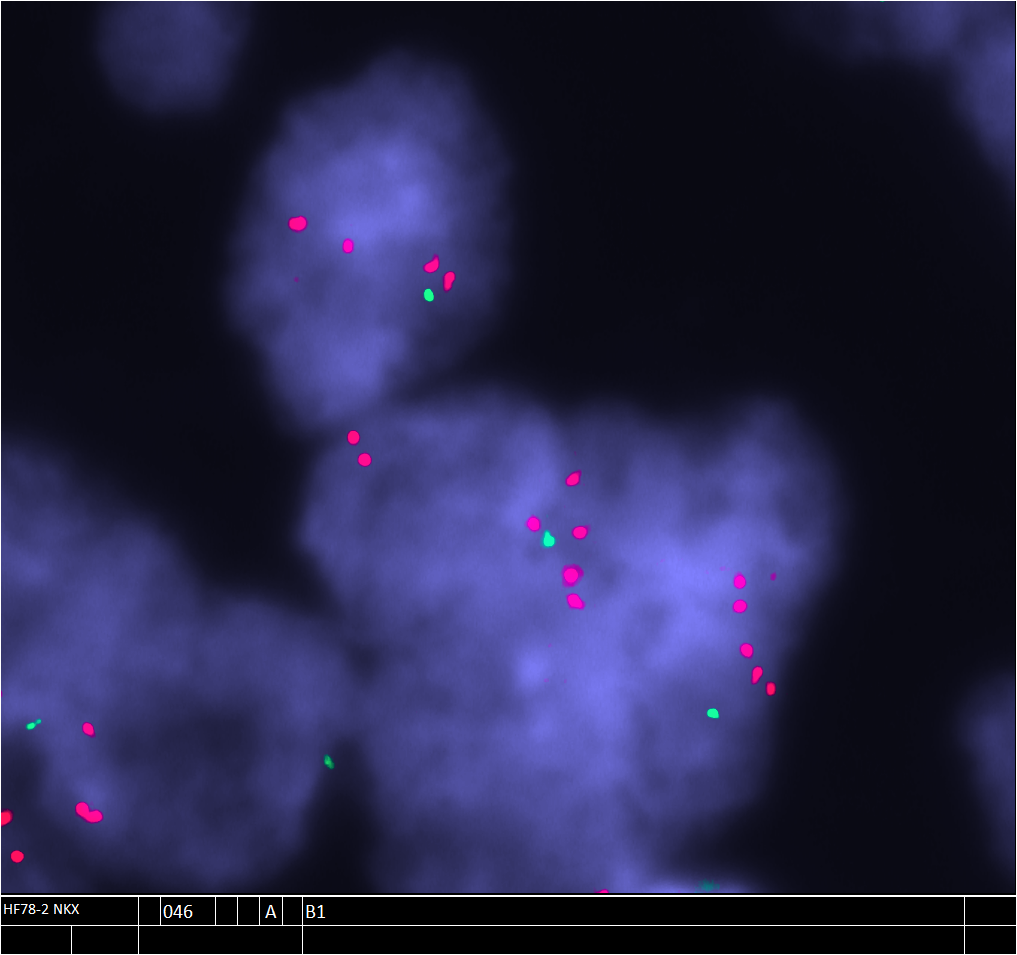

Supplement: Supplementary file 1 [file cancers-13-02363-s001.zip › cancers-1141750 - supple.- revised version/ME-S1 Figure.TIF]
